# Influence of Ethanol as a Preservative in Topical Formulation on the Dermal Penetration Efficacy of Active Compounds in Healthy and Barrier-Disrupted Skin

**DOI:** 10.3390/pharmaceutics17020196

**Published:** 2025-02-04

**Authors:** Christian Raab, Tien Trung Do, Cornelia M. Keck

**Affiliations:** Department of Pharmaceutics and Biopharmaceutics, Philipps University of Marburg, Robert Koch Str. 4, 35037 Marburg, Germany

**Keywords:** preservative, skin, ethanol, dermal penetration, barrier-disrupted skin, healthy skin

## Abstract

(1) Background: Ethanol is a multifunctional excipient often used as a preservative in topical formulations. Due to its known ability to impair skin barrier function, this study investigated the effect of ethanol (EtOH) as a preservative in creams on the dermal penetration of active compounds. (2) Methods: A hydrophilic and a lipophilic fluorescent dye were used as active ingredient surrogates that were incorporated into creams with and without ethanol. The dermal penetration efficacy was assessed by epifluorescence microscopy on an ex vivo porcine ear model with intact and irritated skin. (3) Results: Ethanol reduced the dermal penetration by about 40% for the hydrophilic and about 20% for the lipophilic surrogates on intact skin, but had minimal impact on irritated skin. The bio-physical skin properties were also altered by the addition of ethanol to the cream. On intact skin, it increased transepidermal water loss (TEWL) and decreased skin hydration, whereas on irritated skin, it decreased TEWL and increased skin hydration. The results indicate that skin impairment can be considered to have different stages, while in an early stage of skin impairment, the formation of a “Pudding skin” is proposed. A “Pudding skin” is the formation of a thin layer of dried skin on top of the skin that “seals” the lower parts of the skin and reduces dermal penetration and water loss from inside the skin and reduces the dermal penetration of chemical compounds from outside the skin. (4) Conclusions: Overall, the findings emphasize the need to carefully consider the use of ethanol in formulations, balancing its preservative benefits with its potential to impair the efficacy of active ingredients, particularly in varying skin conditions.

## 1. Introduction

The topical application of active compounds is often conducted by using semi-solid formulations. This can include creams, gels, lotions, ointments or other types of vehicles in which the active ingredients are incorporated. If these vehicles contain water in their outer phase, these formulations need to be preserved by using preservatives. Classical examples of preservatives that are used in pharmaceutical and cosmetic formulations include parabens, benzoic acid, sorbic acid and various types of alcohols [1,2,3,4,5,6]. The addition of preservatives to topical formulations is critical, as many preservatives can cause incompatibilities or instability issues [6,7,8,9]. Therefore, a thoughtful selection of a suitable preservative that allows for effective antimicrobial treatment and maintenance of chemical and physical stability at the same time is needed for each individual formulation.

EtOH, one of the most common of the natural preservatives, acts as such when added in concentrations above 15% to the water phase of a formulation, and is considered to be safe and non-toxic [10]. Despite its preservative properties, ethanol is known to possess several other properties. For example, it acts as a solvent and penetration enhancer [2,11,12,13,14,15,16,17,18,19,20,21]. The latter effect is concentration-dependent and is considered to occur due to a washout effect of free fatty acids from the stratum corneum and by increasing the lipid chain movements within the stratum corneum lipids, which alters the bilayer structure of the stratum corneum [22]. Based on the above-mentioned changes in the skin barrier, we also know a damaging effect of EtOH, where higher dosage is considered to damage the skin or lead to systemic applications [11,15,21].

We hypothesized that the use of EtOH as a preservative in topical formulations may alter the dermal penetration of active ingredients in topical formulations and, along with this, also the bio-physical skin properties after the application of these topical formulations. Most of the studies use pure ethanol or related alcohols, as well as mixtures of water and ethanol to investigate penetration effects [16].

This study investigated a more complex system by formulating a commercially available cream with and without EtOH as a preservative. The influence of the EtOH on dermal penetration efficacy was tested for hydrophilic and lipophilic active compounds, with an ex vivo porcine ear model with subsequent image analysis. The bio-physical skin properties were assessed by measuring the transepidermal water loss (TEWL) and the skin hydration after the different treatments. The experiments were performed on healthy, intact porcine skin and also on irritated skin mimicking barrier-disrupted conditions. Differentiating between these skin conditions is important to understand how EtOH, as a preservative, influences formulations, especially in products intended for sensitive or barrier-disrupted skin.

## 2. Materials and Methods

### 2.1. Materials

Sodium fluorescein (Carl Roth GmbH & Co. KG, Karlsruhe, Germany), a hydrophilic fluorescent dye, was used as a surrogate for a hydrophilic active pharmaceutical/cosmeceutical ingredient (API), and Nile red (Sigma-Aldrich Chemie GmbH, Steinheim, Germany), a lipophilic fluorescent dye, was used as a surrogate for a lipophilic API. The compositions and sources of the ingredients in the creams, both with and without EtOH as a preservative, are provided in Table 1 and Table 2. The purified water for the production of the formulations was freshly obtained in-house from a PURELAB^®^ Flex 2 (ELGA LabWater, Veolia Water Technologies GmbH, Celle, Germany). Prior its use, the purified water was decontaminated by boiling it for 5 min [23]. After boiling, it was allowed to cool down to room temperature and was then used immediately.

### 2.2. Methods

#### 2.2.1. Production of Topical Formulations

The creams were prepared by the hot emulsification method. For this, the water phase and the oil phase were heated to 70 °C separately and were then combined by emulsifying the oil phase into the water phase using a reaction chamber (LR 250 IKA-Laborreaktor, IKA Werke GmbH & Co. KG, Staufen, Germany) at 50 rpm. For the cream being preserved with 20% (*v*/*v*) EtOH, the EtOH was added to the water phase only shortly prior to the addition of the oil phase. The API surrogates were added to the freshly prepared creams as either oily or aqueous stock solutions by using mortar with a pestle. The final formulations contained either 0.005% *w*/*w* sodium fluorescein or 0.005% *w*/*w* Nile red as a hydrophilic or lipophilic API surrogate, respectively.

#### 2.2.2. Characterization of the Topical Formulations

The creams obtained were characterized by their most relevant physicochemical parameters to understand the effect of the EtOH incorporated into the formulations. Hence, their particle size, size distribution and physical stability were assessed using laser diffraction (LD), inverted epifluorescence microscopy and centrifugation.

LD measurements were performed with a Mastersizer 3000 (Malvern-Panalytical, Kassel, Germany). The measurements were conducted using a HydroS dispersion unit at a stirring speed of 1700 rpm, without sonication, and the results were analyzed using Mie theory with 1.45 as a real refractive index and 0.01 as an imaginary refractive index. The particle sizes were determined as volume-based median diameters (d(v)-values), where the size given represents the maximum particle size for the specified volume. For example, a d(v) 0.5 of 1 µm represents that 50% of the volume of the particles is ≤1 µm.

Inverted epifluorescence microscopy (OLYMPUS BX53, Olympus Deutschland GmbH, Hamburg, Germany) was used to visualize the cream structure, the droplet size and the size distribution. In addition, it was used to visualize the distribution of the API surrogates within the creams.

The physical stability of the formulations was assessed by centrifugation (Hettich Mikro 120, Andreas Hettich GmbH & Co. KG, Tuttlingen, Germany, 4000 rpm, 10 min) and by assessing the degree of phase separation after the centrifugation. For this, the total height of 1 mL formulation in an Eppendorf tube was measured and the height of the separated oil phase after centrifugation. The relative height of the separated oil phase was then calculated and used to represent the degree of instability of the emulsion [24,25].

#### 2.2.3. Determination of Dermal Penetration Efficacy

The dermal penetration efficacy was assessed using the ex vivo porcine ear model, followed by digital image analysis [26,27]. Fresh ears were obtained from a local slaughterhouse and used within 2 h after slaughter. Before use, the ears were carefully washed with lukewarm water and dabbed dry with a soft tissue. Afterwards, the ears were divided into two groups: Group I, representing intact skin, which underwent no further treatment, and Group II, representing irritated skin, which was treated with acetone to alter its physiological condition, following a previously established protocol [28]. Accordingly, to mimic irritated and barrier-disrupted skin, the ears were dipped into acetone for 30 s and then let to dry for 10 min. The procedure was performed twice.

On the ready prepared ears, areas with a size of 2 cm × 2 cm, free of visible damages, were marked, and the hair was trimmed to 1–2 mm. Next, 4 mg of each formulation (corresponding to a dose of 1 mg/cm^2^—mimicking a realistic dose in real-life applications) was applied on the prepared areas and distributed with a pipette tip without pressure. The penetration time was 2 h at 32 °C. After the penetration time, the ears were removed from the oven and formulation residues were carefully removed with a wet soft paper towel. Punch biopsies (Ø 15 mm) were then taken from the treated skin areas, immediately embedded in TissueTek^®^ (Sakura Finetek Europe B.V., Alphen aan den Rijn, The Netherlands) and frozen at −30 °C until further use.

From each frozen skin biopsy, 15 vertical, 20 µm thin skin sections were obtained using a cryomicrotome (Frigo-Cut 2700, Reichert, Germany), and images were obtained using inverted epifluorescence microscopy (Olympus Deutschland GmbH, Hamburg, Germany). The filter block system FITC (excitation filter: 467–498 nm (BP), dichroic mirror 500 nm, emission filter: starting at 513 nm (LP)) was used for the hydrophilic API surrogate and the TRITC setup (excitation filter: 532–554 nm (BP), dichroic mirror 562 nm, emission filter: starting at 570 nm (LP)) was used for the lipophilic API surrogate. For all images, the exposure time was set to 50 ms, and the intensity of the fluorescent light source (130 W U-HGLGPS illumination system, Olympus Deutschland GmbH, Hamburg, Germany) to 50%. The magnification was 200-fold. Each sample was tested in triplicate and on three independent ears (ears from different donor pigs). From each skin biopsy, 40 images were obtained, resulting in a total of 120 images per sample.

The obtained fluorescence images were analyzed to determine the amount of penetrated API surrogate (ART), the mean penetration depth (MPD) and the stratum corneum thickness (SCT) using ImageJ software [29]. The ART was determined by applying an automated RGB macro (c.f. Supplementary Materials S1) to the images, which subtracted the autofluorescence of the skin from the images. The remaining pixels after the RGB threshold (ART) in each image were then measured as the mean gray value per pixel (MGV/px) and represent a semi-quantitative measure of the amount of penetrated active ingredients into the skin [27]. The MPD was measured from the digitally processed images by measuring the distance from the skin surface to the most distant pixel in each image. Measurements were taken manually using the scale function of the Image J software. The SCT was similarly determined but from the original images [27].

#### 2.2.4. Determination of Bio-Physical Skin Parameters

As bio-physical skin parameters, the transepidermal water loss (TEWL) and skin hydration were measured. The TEWL was assessed using the Tewameter^®^ TM 300 (Courage & Khazaka electronic GmbH, Cologne, Germany), which measures the water loss through the skin, providing an indication of the integrity of the skin barrier. The skin hydration was measured by assessing the skin capacitance on top of the skin by using the Corneometer^®^ CM 825 (Courage & Khazaka electronic GmbH, Cologne, Germany). In addition, the MoistureMap MM200 (Courage & Khazaka electronic GmbH, Cologne, Germany) was used to measure and to visualize the distribution of the moisture across the skin surface.

#### 2.2.5. Statistical Analysis

All data are presented as the mean ± standard deviation (SD), with each sample tested in triplicate using ears from three independent donor pigs. Statistical analysis was performed using JASP software (version 0.17.3.0, JASP Team, Amsterdam, The Netherlands) [30]. The normal distribution of the data was assessed with the Shapiro–Wilk test, and variance homogeneity was evaluated using the Levene’s test. One-way analysis of variance (ANOVA) with Welch adoption in case of variance inhomogeneity was conducted for normally distributed data and Kruskal–Wallis H tests were performed for non-parametric data sets. Appropriate post hoc tests (Tukey, Games–Howell or Dunn’s post hoc test) were also performed to determine significant differences between the various mean values. Probability values (*p*-values) of <0.05 were considered statistically significant.

## 3. Results and Discussion

### 3.1. Production and Characterization of Creams with and Without EtOH

**Figure 1:**
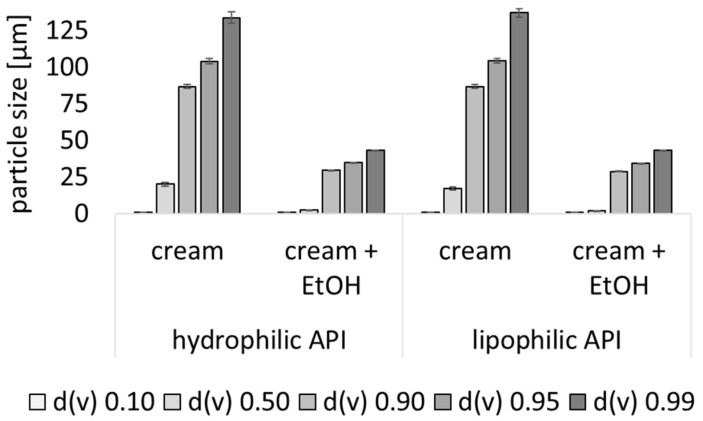
Particle size analysis of creams with and without EtOH (LD data).

**Figure 2:**
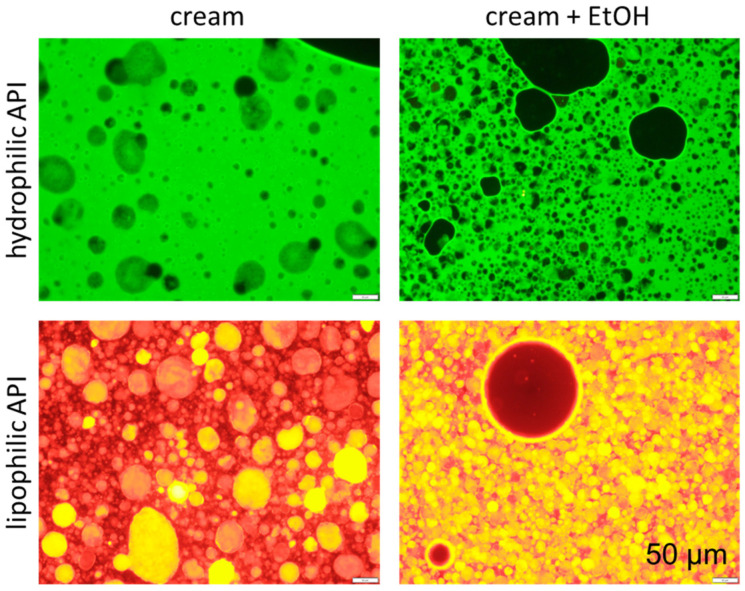
Fluorescence microscopic images of creams with and without EtOH loaded with either hydrophilic API surrogate (**upper**) or lipophilic API surrogate (**lower**). A 200-fold magnification was used and the scale bar represents 50 µm.

The freshly prepared creams without EtOH possessed a mean particle size (d(v) 0.5) of about 20 µm and a relatively broad particle distribution, with sizes ranging from about 1 µm (d(v) 0.1) to about 130 µm (d(v) 0.99). The preservation of the creams with EtOH decreased the mean particle size to about 2 µm (d(v) 0.5) and narrowed the particle size distribution (Figure 1). The size-reducing effect of EtOH observed by the LD measurements was also confirmed by fluorescence microscopy, which clearly showed much smaller oil droplets when EtOH was added to the creams (Figure 2). In addition, the fluorescence microscopy images also showed that the hydrophilic API surrogate was dissolved in the outer phase of the cream, whereas the lipophilic API surrogate was dissolved in the inner, oily phase of the o/w creams.

**Figure 3:**
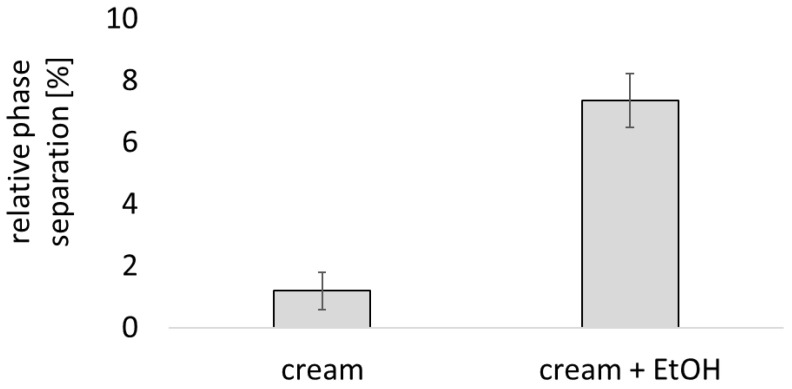
Influence of EtOH on physical stability of cream, assessed as relative phase separation after 10 min centrifugation at 4000 rpm.

Typically, smaller sized oil droplets lead to formulations with improved physical stability when compared to formulations that contain larger droplet sizes [31]. Hence, the data suggested that the addition of EtOH caused an increased physical stability of the cream. To check for this assumption, a stress test was performed. For this, the formulations were centrifuged and the degree of phase separation that occurred during the centrifugation was assessed (Figure 3). The phase separation was <2% for the creams without EtOH but >7% for the creams with added EtOH, clearly showing that the addition of EtOH caused a decreased physical stability of the creams. Hence, the formulations that contained EtOH possessed a smaller particle size but were less physically stable than the creams without EtOH.

### 3.2. Influence of EtOH on Dermal Penetration Efficacy

**Figure 4:**
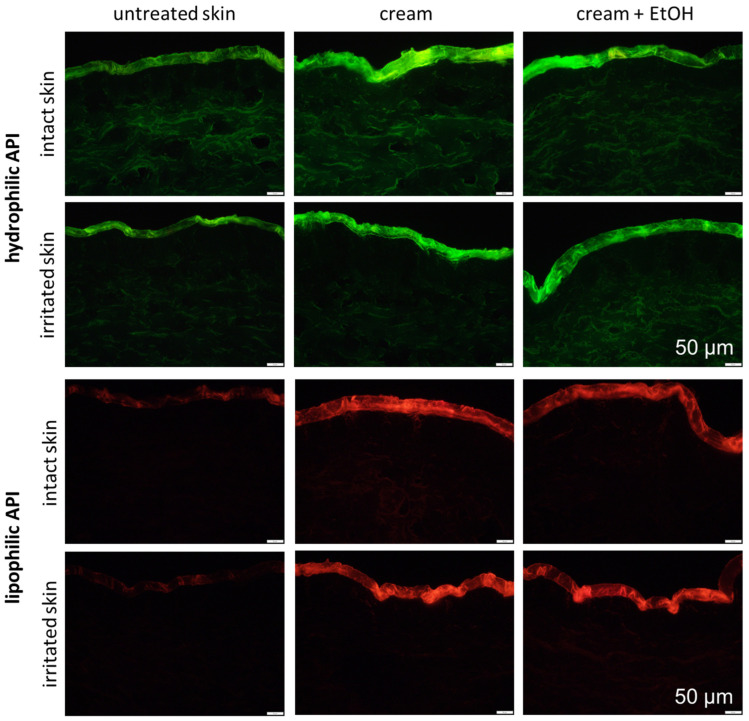
Vertical skin cuts obtained from inverted epifluorescence microscopy of intact and irritated skin, visualizing the influence of skin treatment with cream and cream preserved with EtOH (20% *v*/*v*) on the dermal penetration efficacy of hydrophilic and lipophilic active API surrogates on intact and irritated skin. (200-fold magnification, scale represents 50 µm).

**Figure 5:**
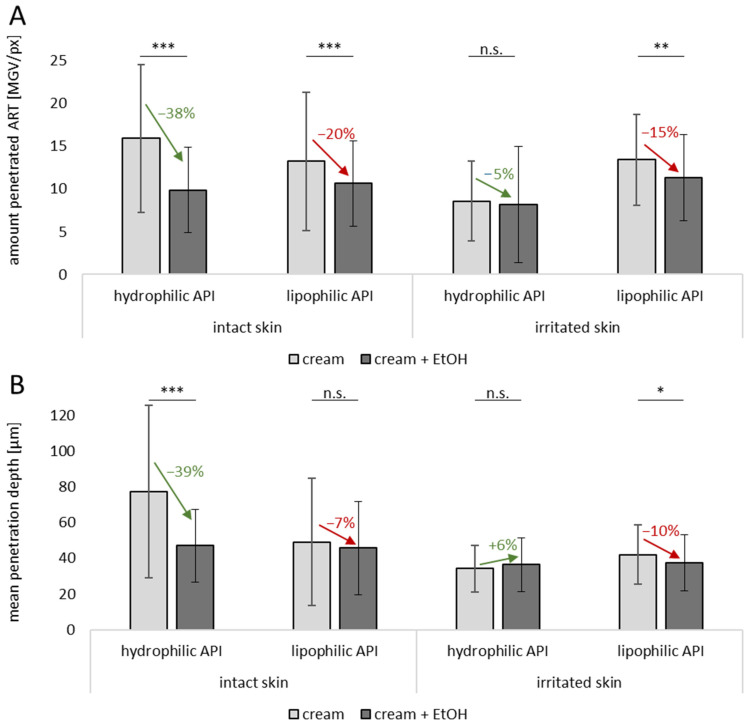
Influence of skin treatment with cream and cream preserved with EtOH (20% *v*/*v*) on dermal penetration efficacy of hydrophilic and lipophilic active API surrogates on intact and irritated skin. (**A**) Amount of penetrated API (ART), (**B**) mean penetration depth (MPD). n.s.—non significant, * ρ < 0.05, ** ρ < 0.01, *** ρ < 0.001.

The influence of EtOH on the dermal penetration efficacy was tested for a hydrophilic and a lipophilic API surrogate on intact and irritated, barrier-impaired skin. The images obtained from the vertical skin cuts already indicate that EtOH decreases the penetration efficacy for both types of API (Figure 4), and the data obtained from digital image analysis confirm this (Figure 5). The effect was more pronounced for the hydrophilic API surrogate, where the presence of EtOH decreased the amount of penetrated API surrogate and the mean penetration depth by almost 40% (Figure 5). For the lipophilic API surrogate, the addition of EtOH reduced the amount of penetrated API surrogate by about 20%, and the mean penetration depth was reduced by about 7% (*p* > 0.5%). The effects observed were not expected, because in general, EtOH is known to enhance the dermal penetration of active compounds [10]. Although the penetration-enhancing effects are typically associated with absolute or highly concentrated EtOH, our study used a 20% (*v*/*v*) EtOH concentration, and EtOH was incorporated into a complex formulation. Consequently, the observed effects may differ from those reported in studies using higher EtOH concentrations [22].

Irritated skin is characterized by an impaired skin barrier. As EtOH can also be used as a preservative for formulations that are applied on sensitive skin or skin with an impaired skin barrier, it was also interesting to test the effect of EtOH on the dermal penetration efficacy of API on this type of skin. The images of the vertical skin cuts show almost no differences for the dermal penetration efficacy of the API from the formulations with and without EtOH (Figure 4), and the data obtained from digital image analysis confirmed this (Figure 5). EtOH caused no significant changes in the penetration efficacy of the hydrophilic API surrogate (no significant changes in ART values and no significant changes in the MPD). For the lipophilic API surrogate, a slight decrease was observed. The total amount of penetrated API was decreased by 15% and the penetration depth was decreased by 10%. These effects were also small but significant. Also, these effects were not expected, because EtOH—with its known penetration-enhancing effects—was expected to impair the skin barrier and thus to promote the penetration of active compounds into the skin. This barrier-impairing and penetration-enhancing effects were expected to be even more pronounced on irritated skin with an already impaired skin barrier. However, opposite effects were observed in this study.

Interestingly, it was also found that the dermal penetration efficacy of the hydrophilic API surrogate on irritated skin was decreased by 46% when compared to the intact skin (Figure 5). However, no differences in the penetration efficacy were observed for the lipophilic API surrogate, meaning that similar penetration profiles were obtained for the intact and irritated skin (Figure 5).

Based on the data, it can be concluded that EtOH has different (opposite) effects on the penetration efficacy of hydrophilic and lipophilic APIs. Similar differences in penetration efficacy between hydrophilic and lipophilic APIs were observed in a previous study, which showed that APIs can penetrate the skin via a solvent drag mechanism. Solvent drag mechanism means that liquids can penetrate into the skin, and the APIs that are dissolved in these liquids will be dragged into the skin together with the solvent [32]. In the before mentioned study, the penetration of the API was linked to skin hydration and a higher skin hydration was associated with a higher penetration of the hydrophilic API and vice versa.

In the present study, acetone was used to impair the skin barrier to mimic irritated skin, and EtOH was used as a preservative. Both chemicals are known to damage and dehydrate the skin [33,34]. Therefore, it seems to be likely that the changes in dermal penetration efficacy for both compounds are due to changes in skin hydration upon treatment with acetone and/or EtOH. Therefore, the next step aimed at investigating the influence of EtOH and acetone on the skin barrier function and skin hydration in more detail.

### 3.3. Bio-Physical Skin Parameters

#### 3.3.1. Barrier Integrity

**Figure 6:**
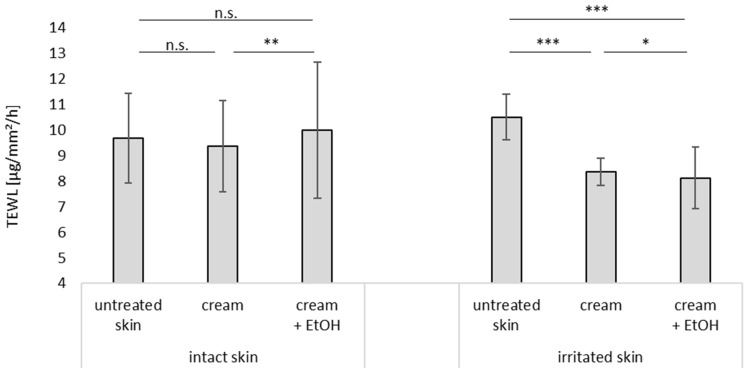
Influence of skin treatment with cream and cream preserved with EtOH (20% *v*/*v*) on TEWL of intact and irritated skin. n.s.—non significant, * ρ < 0.05, ** ρ < 0.01, *** ρ < 0.001.

The skin barrier integrity was measured by determining the TEWL, which indicates the water loss from the skin over time (Figure 6). For human skin, the TEWL greatly varies from <3 to >40 g/m^2^/h and depends on many parameters [35]. The intact skin of porcine ears with a non-impaired stratum corneum typically should possess a TEWL < 10 g/m^2^/h, and hence, impaired skin can be recognized by a TEWL > 10 g/m^2^/h [26,28]. The TEWL of the intact skin was 9.7 g/m^2^/h in our study. It slightly decreased upon the application of the cream (*p* > 0.5) and increased when EtOH was added as a preservative, indicating a slight skin barrier-interrupting effect of the EtOH. The TEWL of the irritated skin was >10 g/m^2^/h and decreased to below 8.4 g/m^2^/h upon the application of the cream, indicating that the cream had a barrier-protecting effect on the irritated skin. The addition of the EtOH led to a further decrease in the TEWL. Hence, the barrier-irritating effect of the cream with EtOH on intact skin was not observed for the irritated skin. This suggests that, on irritated skin, EtOH had no negative effect on the skin barrier integrity.

#### 3.3.2. Skin Hydration

**Figure 7:**
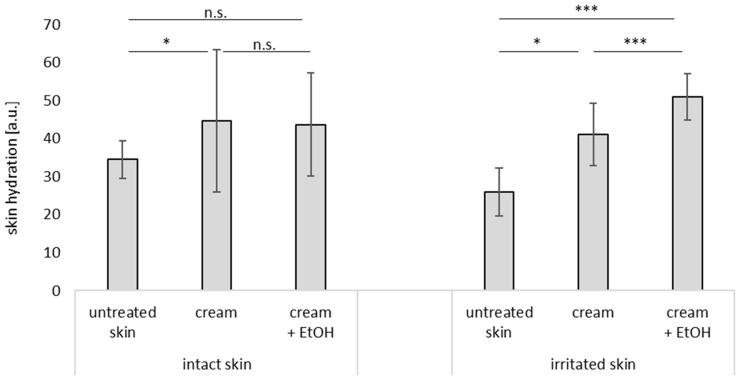
Influence of skin treatment with cream and cream preserved with EtOH (20% *v*/*v*) on skin hydration (Corneometer data) of intact and irritated skin. n.s.—non significant, * ρ < 0.05, *** ρ < 0.001.

**Figure 8:**
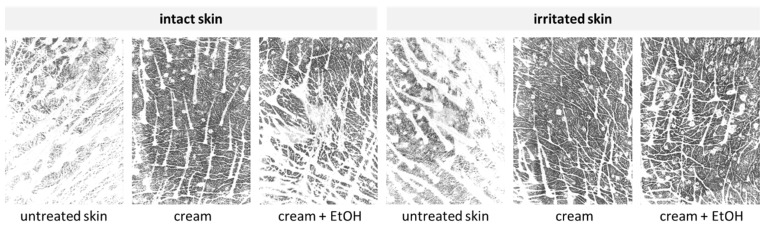
Images of porcine skin obtained with a MoistureMap visualizing the influence of skin treatment with cream and cream preserved with EtOH (20% *v*/*v*) on skin moisture.

**Figure 9:**
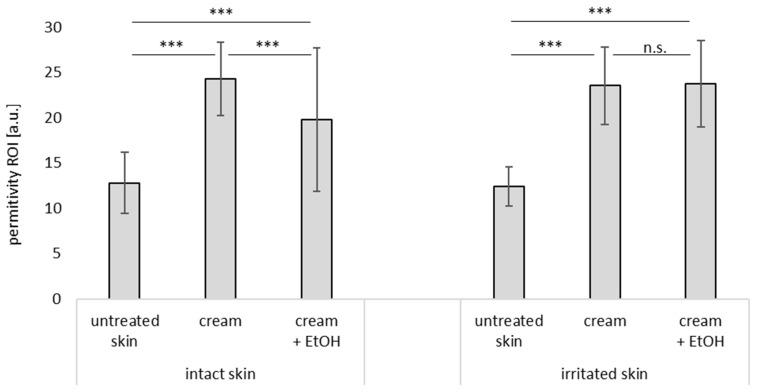
Influence of skin treatment with cream and cream preserved with EtOH (20% *v*/*v*) on skin moisture (MoistureMap data) of intact and irritated skin. n.s.—non significant, *** ρ < 0.001.

The skin hydration on top of the skin was measured with two different techniques (Figure 7, Figure 8 and Figure 9). Both instruments measure the capacitance of the skin surface and higher values represent a higher moisture of the skin surface. For both types of measurements, skin hydration increased upon the application of the cream. When EtOH was added to the cream, the increase in skin hydration after the application of the cream was less pronounced than it was with the cream without EtOH, thus confirming the skin-dehydrating effect of EtOH.

When compared to the untreated intact skin, the skin hydration of the untreated irritated skin was lower. The application of cream to the irritated skin increased skin hydration, as similarly seen for the intact skin. However, cream with EtOH did not cause a dehydrating effect as was seen for the intact skin, but led to increased skin hydration instead. These findings are in line with the TEWL data, which already indicated that the addition of EtOH to the cream caused a slight dehydrating and barrier-disrupting effect on the intact skin, but had no irritating effects on the already irritated skin (c.f. Figure 6).

#### 3.3.3. Stratum Corneum Thickness

**Figure 10:**
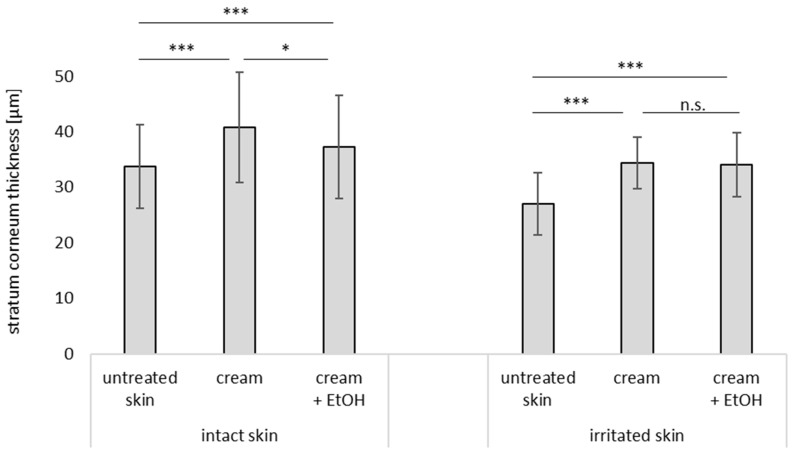
Influence of skin treatment with cream and cream preserved with EtOH (20% *v*/*v*) on stratum corneum thickness of intact and irritated skin. n.s.—non significant, * ρ < 0.05, *** ρ < 0.001.

The stratum corneum thickness can be obtained from the vertical skin cuts and provides additional valuable information on skin hydration. In contrast to the capacitance measurements, that give only information on the moisture on top of the skin, the skin cuts can provide information on the hydration state of the entire stratum corneum. The thicker the stratum corneum, the more moisture is captured within the stratum corneum. The stratum corneum of untreated intact skin was 34 µm and increased by about 20% to 41 µm after treatment with the cream. Upon the treatment with cream that contained EtOH, the increase was only 10% (Figure 10). The stratum corneum of the untreated irritated skin was 27 µm thick, which corresponds to about 80% of the SCT of the intact skin. Hence, the SCT of irritated skin was decreased by 20% when compared to the healthy, intact skin. Upon treatment with cream, the thickness of the irritated skin increased by about 27% to 34 µm when cream with and without EtOH was applied to it. Hence, the hydration effect of the cream on the irritated skin was more effective than it was for the intact skin. In addition, also these data show that the EtOH that was added to the cream as a preservative had a slight barrier-impairing and dehydrating effect on intact skin, but had not such effects on irritated skin.

### 3.4. Synopsis of Data

Based on the results obtained from our study, the following conclusions can be drawn:

1. Treating intact skin with an o/w cream increased the skin hydration, had no impact on the TEWL and allowed for a good dermal penetration for the hydrophilic and lipophilic active ingredient surrogates.
2. Treating intact skin with an o/w cream that contained EtOH as a preservative decreased the skin hydrating effect of the cream, increased the TEWL and decreased the dermal penetration.
3. When compared to intact skin, irritated skin was characterized by a higher TEWL, higher skin hydration on top of the skin but reduced stratum corneum thickness, which indicates an all-over lower skin hydration. These skin properties resulted in a less pronounced dermal penetration efficacy for active compounds when compared to intact skin.
4. Treating irritated skin with the o/w cream increased the skin hydration more efficiently than on intact skin and decreased the TEWL of the skin.
5. Treating irritated skin with the o/w cream that contained EtOH as a preservative decreased the TEWL and increased the skin hydration. The dermal penetration efficacy was not changed for the hydrophilic active ingredient surrogate and was slightly decreased for the lipophilic active ingredient surrogate.

The synopsis of the data shows that an increase in TEWL and a decrease in skin hydration (both observed in intact EtOH-treated skin and irritated skin) were associated with a decrease in dermal penetration efficacy. This is sensible, because a higher TEWL means that more water evaporates from the inside to the outside of the skin. The evaporating water, that travels the skin from the inside to the outside, has an opposite flow direction than the molecules that aim to penetrate into the skin. Therefore, a high TEWL can hamper the penetration of active compounds into the skin. In addition, dry skin can be considered to be tighter. Hence, the intercellular space between the corneocytes is narrower and consequently, the diffusion coefficient for active molecules that aim to penetrate into the skin will be lower, which then results in a decreased dermal penetration efficacy.

Irritated skin and EtOH are known to cause dry skin with increased TEWL values but are generally considered to increase the dermal penetration efficacy of active compounds. The considered increase in dermal penetration efficacy is explained by a distortion of the skin barrier, which results in a disrupted stratum corneum structure that is more permeable for molecules to enter the skin. Our study confirmed the increased TEWL and decreased skin hydration for both the irritated skin and the EtOH. However, the penetration-enhancing effects could not be confirmed in our study. The explanation for the decreased dermal penetration in our study is still very reasonable and indicates that the skin barrier of the irritated skin in our study—that is also characterized by dryness and increased TEWL values—seems to have no disrupted stratum corneum, i.e., “holes” in it that increase the permeability of compounds into the skin. Our data rather show that dry skin with a high TEWL seems to be even protective against the penetration of active compounds.

**Figure 11:**
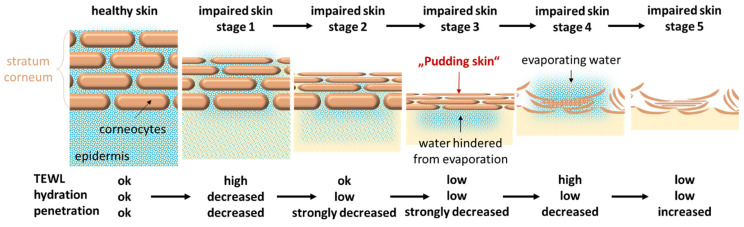
Scheme of different stages of impaired skin with associated skin properties and predicted penetration efficacies for chemical compounds into the skin.

Therefore, as a hypothesis, it might be assumed that impaired skin can have different stages of impairment and barrier disruption (Figure 11). At early stages of impaired skin, the skin has high TEWL values, which reduces the skin hydration over time (Figure 11—stage 1). As the water evaporates from the surface, the skin will become dryer from the surface but will remain hydrated in lower skin layers (Figure 11—stage 2). With further drying, the dehydration of the upper skin layers will continue and result in a sealing (self-healing) effect of the skin (Figure 11—stage 3). This could be considered to be similar to the thin skin that forms on top of a pudding when it is not stirred during cooling. The “Pudding skin” of the skin prevents further unhindered water loss from the skin which is—as it cannot evaporate anymore—retained below the dried skin layer. The dry skin layer is denser than hydrated skin, so increased dryness reduces the dermal penetration of chemicals from the outside to the inside of the skin. However, the “Pudding skin”—due to its dryness—is less elastic and more brittle than normally hydrated skin. The brittleness can be considered to increase with the ongoing dryness of the skin and thus will certainly lead to ruptures and cracks within the skin (Figure 11—stage 4). These ruptures then create “holes” in the skin that will—most likely—increase the TEWL as the retained water below the dry skin layer can now be released to the skin surface from where it will evaporate. During this period, the evaporating water from the skin moves from the inside to the outside of the skin and thus creates a push effect which will further hinder the penetration of chemicals from the outside to the inside of the skin. With the ongoing evaporation of water, the skin will completely dry out and the TEWL of the skin will decrease. At this stage, the skin barrier is strongly impaired and dry, and chemical compounds can be considered to easily penetrate into the skin (Figure 11—stage 5).

**Figure 12:**
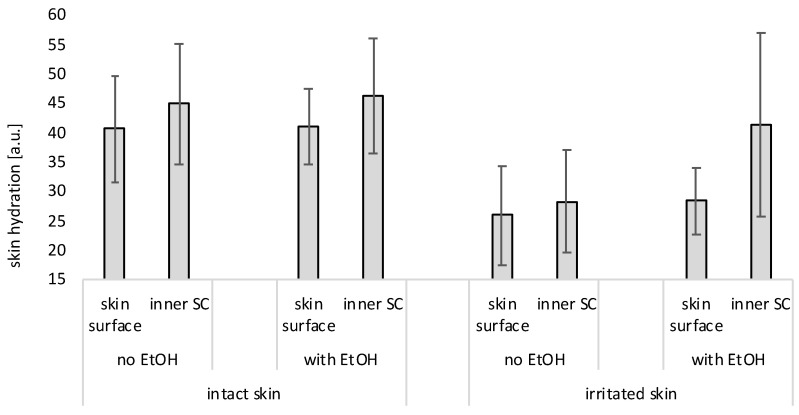
Influence of EtOH (20% *v*/*v*) on skin hydration of intact and irritated skin. The skin hydration was measured on top of the skin (skin surface) and in the inner stratum corneum (inner SC) after removing parts of the stratum corneum by classical tape stripping.

The hypothesis of the different stages of impaired skin conditions, where TEWL and skin hydration are differently correlated to each other, is supported by various studies that already showed the controversy of TEWL measurements and skin hydration [36,37]. However, a study that investigates the skin hydration in different layers of the skin and thus would allow for a visualization of the proposed “Pudding skin effect” was not available. Therefore, such an experiment was performed as a final step in this study. For this, intact and irritated skin were treated with 20% (*v*/*v*) EtOH. The skin hydration was measured after 1 h on top of the skin and after removing 30 layers of the stratum corneum by the classical tape stripping procedure [28,38]. The results obtained were compared to untreated skin (Figure 12).

On intact skin, the skin hydration within the stratum corneum (after tape stripping) was about 10% higher than on top of the skin. When the intact skin was treated with EtOH, skin hydration within the stratum corneum was 13% higher than the hydration on top of the skin. The effects were not significant but show the expected trend that water is retained in deeper skin layers when a thin and dry skin layer (“Pudding skin”) is formed on top of the skin.

The inner and outer skin hydration of the non-treated irritated skin was decreased by about one third, when compared to the intact skin. However, the difference in skin hydration between the inner and outer skin was similar to that of intact skin (approx. −10%). The treatment of the irritated skin with EtOH resulted in a small increase in skin hydration on the surface (approx. +10%), but increased the hydration of the inner stratum corneum by almost 50%. Hence, the treatment of the irritated skin with EtOH solution could almost restore the inner skin hydration of non-irritated skin, strongly supporting the formation of a “Pudding skin” after the application of EtOH on the irritated skin.

**Figure 13:**
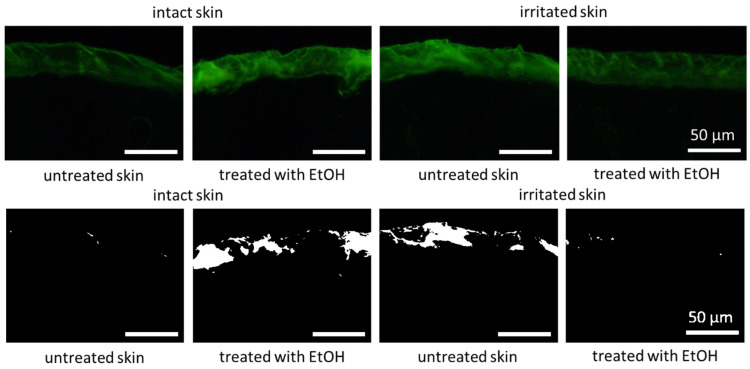
Vertical skin cuts obtained from inverted epifluorescence microscopy of intact and irritated skin—either untreated or treated with 20% (*v*/*v*) EtOH solution. (**Upper**) digital zooms of original images (200-fold magnification), (**lower**) black and white images of the original zooms.

The restoring and “self-healing” effect of the EtOH treatment on the irritated skin was also visible on the skin cuts that were obtained from the differently treated skin areas (Figure 13-upper). The autofluorescence of the stratum corneum of the untreated intact skin was relatively low, which indicates a good skin hydration throughout the entire stratum corneum [39]. After the application of EtOH in the intact skin, the autofluorescence of the stratum corneum appeared higher, which consequently indicates decreased skin hydration. The autofluorescence of the irritated skin was also high but decreased after the treatment of the skin with EtOH, indicating that the EtOH treatment indeed could increase the skin hydration—most likely due to the formation of the “Pudding skin”. The effects described became even more visible after converting the fluorescence microscopic images into a black and white format (Figure 13-lower).

The results of the “Pudding skin experiment” could clearly demonstrate the increased skin hydration in the lower stratum corneum upon the treatment of irritated skin with 20% EtOH solution. These preliminary data therefore provide first evidence for the formation of a “Pudding skin” on top of the skin and the proposed sealing (self-healing) effect of the skin. A missing link between the SCT, skin hydration and dermal penetration efficacy would have been the assessment of the viscoelasticity of the skin. This would have provided a more holistic understanding for the presented hypothesis. A systematic study that investigates these effects is now needed to further explain these findings and their impact on skin properties and dermal penetration efficacy in more detail.

## 4. Conclusions

The study demonstrates the influence of EtOH that was added as a preservative to a cream on the formulation stability, skin properties and dermal penetration efficacy of chemical compounds. The addition of EtOH decreased the particle size of oil droplets and impaired the cream’s physical stability. The dermal penetration efficacy was strongly reduced when the EtOH-preserved formulation was applied to intact skin, but EtOH had almost no effect on irritated skin. On intact skin, the EtOH-containing cream showed a slight barrier-impairing and dehydrating effect, while on irritated skin, a hydrating effect was observed. The results obtained were not expected and may be explained by considering irritated skin not only as dry skin with a disrupted barrier but as skin undergoing different stages of impairment. Early impairment begins with increased water evaporation, leading to high TEWL values and reduced skin hydration. The decreased skin hydration at the surface leads to drying of the upper skin layers, resulting in the formation of a “Pudding skin”—a tighter packing of corneocytes and increased density of the stratum corneum. This reduces the permeability and penetration of active ingredients. Further drying makes the skin brittle, eventually causing ruptures. These ruptures increase the TEWL, dehydrate deeper skin layers, and will allow molecules to penetrate the skin more easily. Similar effects were observed on irritated skin, which allowed less penetration of the active ingredient compared to intact skin.

The data indicate that even small changes in the formulation composition, such as the incorporation of small amounts of EtOH as a preservative, can significantly impact its effects on the skin. This highlights the sensitivity of formulations to ingredient ratios and the importance of precision and acuate testing during development. The findings also suggest that the formulation efficacy varies significantly with skin conditions, emphasizing the need for tailored approaches.

In summary, the choice and concentration of preservatives like EtOH, along with overall formulation composition and skin condition, are crucial to effectiveness, requiring careful customization to optimize therapeutic outcomes for diverse skin types and conditions.

## Figures and Tables

**Table 1:** Chemical compositions of the vehicles used in this study.

Chemical Name/Trade Name	INCI	Provider	Amount (*w*/*w*) (g)
hydrophilic phase			
Dermofeel PA-12	SODIUM PHYTATE	Evonik Operations GmbH, Essen, Germany	0.10
Cosphaderm X34	XANTHAN GUM	Cosphatec GmbH, Hamburg, Germany	0.25
Dermofeel GSC SG	GLYCERYL STEARATE CITRATE	Evonik Operations GmbH, Essen, Germany	3.00
Glycerin 86.5%	GLYCERIN	Fisher Scientific GmbH, Schwerte, Germany	5.00
lipophilic phase			
Dermofeel TOCO 70 non GMO	TOCOPHEROL, HELIANTHUS ANNUUS (SUNFLOWER) SEED OIL	Evonik Dr. Straetmans GmbH, Hamburg, Germany	0.20
Lanette O	CETEARYL ALCOHOL	Caesar & Loretz GmbH, Hilden, Germany	2.00
Sunflower Oil	HELIANTHUS ANNUUS (SUNFLOWER) SEED OIL	Brökelmann & Co, Hamm, Germany	5.00
Palmester 3595	CAPRYLIC/CAPRIC TRIGLYCERIDE	KLK Emmerich GmbH, Emmerich am Rhein, Germany	6.00
Eutanol G	OCTYLDODECANOL	BASF, Ludwigshafen, Germany	6.00

**Table 2:** Chemical composition of the vehicle with ethanol used in this study.

Chemical Name/Trade Name	INCI	Provider	Amount (*w*/*w*) [g]
hydrophilic phase			
Dermofeel PA-12	SODIUM PHYTATE	Evonik Operations GmbH, Essen, Germany	0.10
Cosphaderm X34	XANTHAN GUM	Cosphatec GmbH, Hamburg, Germany	0.25
Dermofeel GSC SG	GLYCERYL STEARATE CITRATE	Evonik Operations GmbH, Essen, Germany	3.00
Glycerin 86.5%	GLYCERIN	Fisher Scientific GmbH, Schwerte, Germany	5.00
lipophilic phase			
Dermofeel TOCO 70 non GMO	TOCOPHEROL, HELIANTHUS ANNUUS (SUNFLOWER) SEED OIL	Evonik Dr. Straetmans GmbH, Hamburg, Germany	0.20
Lanette O	CETEARYL ALCOHOL	Caesar & Loretz GmbH, Hilden, Germany	2.00
Sunflower Oil	HELIANTHUS ANNUUS (SUNFLOWER) SEED OIL	Brökelmann & Co, Hamm, Germany	5.00
Palmester 3595	CAPRYLIC/CAPRIC TRIGLYCERIDE	KLK Emmerich GmbH, Emmerich am Rhein, Germany	6.00
Eutanol G	OCTYLDODECANOL	BASF, Ludwigshafen, Germany	6.00
Ethanol 99.8%	ETHANOL	Fisher Scientific GmbH, Schwerte, Germany	11.57

## Data Availability

The original contributions presented in the study are included in the article, further inquiries can be directed to the corresponding authors.

## Supplementary Materials

The following supporting information can be downloaded at: https://www.mdpi.com/article/10.3390/pharmaceutics17020196/s1.
