# Supplementary material for: Influence of Ethanol as a Preservative in Topical Formulation on the Dermal Penetration Efficacy of Active Compounds in Healthy and Barrier-Disrupted Skin
Source: Pharmaceutics. 2025 Feb 4;17(2):196. doi: 10.3390/pharmaceutics17020196 (PMC11858868; doi:10.3390/pharmaceutics17020196)
Supplement: Supplementary file 1 [file pharmaceutics-17-00196-s001.zip › pharmaceutics-3399022-supplementary.pdf]

pharmaceutics

Supplementary materials: Influence of Ethanol as a Preservative in Topical Formulation on the Dermal Penetration Efficacy of Active Compounds in Healthy and Barrier-Disrupted Skin

Christian Raab, Trung Tien Do and Cornelia M. Keck *

Department of Pharmaceutics and Biopharmaceutics, Philipps University of Marburg, Robert Koch Str. 4, 35037 Marburg, Germany; christian.raab@pharmazie.uni-marburg.de (C.R.); tien.do@pharmazie.uni-marburg.de (T.T.D.)
* Correspondence: cornelia.keck@pharmazie.uni-marburg.de

Supplementary information
Supplementary material S1

Macro 1. Used for hydrophilic APCI

```
// Color Thresholder 1.53e
// Autogenerated macro, single images only!
min=newArray(3);
max=newArray(3);
filter=newArray(3);
a=getTitle();
run("RGB Stack");
run("Convert Stack to Images");
selectWindow("Red");
rename("0");
selectWindow("Green");
rename("1");
selectWindow("Blue");
rename("2");
min[0]=0;
max[0]=255;
filter[0]="pass";
min[1]=135;
max[1]=255;
filter[1]="pass";
min[2]=0;
max[2]=255;
filter[2]="pass";
for (i=0;i<3;i++){
  selectWindow(""+i);
  setThreshold(min[i], max[i]);
  run("Convert to Mask");
  if (filter[i]=="stop")   run("Invert");
}
imageCalculator("AND create", "0","1");
imageCalculator("AND create", "Result of 0","2");
for (i=0;i<3;i++){
  selectWindow(""+i);
  close();
```

```
}
selectWindow("Result of 0");
close();
selectWindow("Result of Result of 0");
rename(a);
// Colour Thresholding-------------
run("Invert");
```

Macro 2. Used for lipophilic APCI

```
// Color Thresholder 1.53e
// Autogenerated macro, single images only!
min=newArray(3);
max=newArray(3);
filter=newArray(3);
a=getTitle();
run("RGB Stack");
run("Convert Stack to Images");
selectWindow("Red");
rename("0");
selectWindow("Green");
rename("1");
selectWindow("Blue");
rename("2");
min[0]=120;
max[0]=255;
filter[0]="pass";
min[1]=0;
max[1]=255;
filter[1]="pass";
min[2]=0;
max[2]=255;
filter[2]="pass";
for (i=0;i<3;i++){
  selectWindow(""+i);
  setThreshold(min[i], max[i]);
  run("Convert to Mask");
  if (filter[i]=="stop")    run("Invert");
}
imageCalculator("AND create", "0","1");
imageCalculator("AND create", "Result of 0","2");
for (i=0;i<3;i++){
  selectWindow(""+i);
  close();
}
selectWindow("Result of 0");
close();
selectWindow("Result of Result of 0");
rename(a);
// Colour Thresholding-------------
run("Invert");
```
